# Lightweight Separable Convolution Network for Breast Cancer Histopathological Identification

**DOI:** 10.3390/diagnostics13020299

**Published:** 2023-01-13

**Authors:** Grace Ugochi Nneji, Happy Nkanta Monday, Goodness Temofe Mgbejime, Venkat Subramanyam R. Pathapati, Saifun Nahar, Chiagoziem Chima Ukwuoma

**Affiliations:** 1 Department of Computing, Oxford Brookes College of Chengdu University of Technology, Chengdu 610059, China; 2 Deep Learning and Intelligent Computing Lab, HACE SOFTTECH, Lagos 102241, Nigeria; 3 School of Computer Science and Engineering, University of Electronic Science and Technology of China, Chengdu 611731, China; 4 Department of Cybersecurity, University of Missouri St. Louis, St. Louis, MO 63121, USA; 5 Department of Information System and Technology, University of Missouri St. Louis, St. Louis, MO 63121, USA; 6 School of Information and Software Engineering, University of Electronic Science and Technology of China, Chengdu 611731, China

**Keywords:** CNN, breast cancer, deep learning, histopathological image, image identification, lightweight network

## Abstract

Breast cancer is one of the leading causes of death among women worldwide. Histopathological images have proven to be a reliable way to find out if someone has breast cancer over time, however, it could be time consuming and require much resources when observed physically. In order to lessen the burden on the pathologists and save lives, there is need for an automated system to effectively analysis and predict the disease diagnostic. In this paper, a lightweight separable convolution network (LWSC) is proposed to automatically learn and classify breast cancer from histopathological images. The proposed architecture aims to treat the problem of low quality by extracting the visual trainable features of the histopathological image using a contrast enhancement algorithm. LWSC model implements separable convolution layers stacked in parallel with multiple filters of different sizes in order to obtain wider receptive fields. Additionally, the factorization and the utilization of bottleneck convolution layers to reduce model dimension were introduced. These methods reduce the number of trainable parameters as well as the computational cost sufficiently with greater non-linear expressive capacity than plain convolutional networks. The evaluation results depict that the proposed LWSC model performs optimally, obtaining 97.23% accuracy, 97.71% sensitivity, and 97.93% specificity on multi-class categories. Compared with other models, the proposed LWSC obtains comparable performance.

## 1. Introduction

Majority of women are affected by breast cancer, which is one of the deadliest and most common type of cancer. In the world of today, about 1 in 8 women maybe diagnosed of breast cancer [1]. In order to fight the disease, it is essential to predict breast cancer risk, and there exist two kinds of breast cancer risk [1]. According to the first category, a person may develop breast cancer within a certain time frame [1]. The likelihood of a high-risk gene mutation is implied by the second type [2]. Breast tumors are unnatural expansion of breast tissue that might show themselves as discharge from a lump or nipple or as a change in the skin’s texture near the nipple. Cancers are uncontrolled cell growth that can spread to other parts of the body through the lymphatic and circulatory systems [3]. After lung cancer, breast cancer is the second most common kind of cancer and the leading cause of cancer mortality in women [3]. The condition has gained in notoriety over the past 50 years, and its prevalence has risen recently.

There are currently few standards for breast cancer screening. The Iranian preventive services working group suggests screening for females between the ages of 45 and 70 but makes no firm recommendations for females older than this. Breast cancer risk assessment can be used to both motivate high-risk women who have not yet had screening and to ensure that those who would not otherwise comply with screening requirements do so. Building cancer prevention and risk reduction strategies can also make use of a statistical model that predicts the risk of breast cancer [4]. The contribution of this study is in two-folds. The first-fold handles the problem of low quality attribute of the image by extracting the visual trainable features of the histopathological image using a contrast enhancement and edge detector algorithms. The second-fold implements a deep learning model of separable convolution layers stacked in parallel with multiple filters of different sizes in order to obtain wider receptive fields. Additionally, the factorization and the utilization of bottleneck convolution layers to reduce model dimension are introduced. This proposed model aim to reduce the number of trainable parameters as well as the computational cost sufficiently with greater non-linear expressive capacity than plain convolutional networks.

This paper will be fashioned with the following sections. Section 2 comprises the literature review of the breast cancer diagnosis; Section 3 details the proposed method and the dataset that will be used to validate the framework. Section 4 presents the experimental findings and model evaluation, and the conclusion is written in Section 5.

## 2. Literature Review

In several earlier researches, the Gail model [5], which is a statistical model that calculates the probability of developing breast cancer has been used. The model integrated different breast cancer risk rates, and different inputs are used to forecast a woman’s risk of acquiring breast cancer using logistics regression [5]. The researchers in [6] utilized the Breast Cancer Risk Assessment Tool also known as BCRAT with the six traditional Gail model inputs plus a typical hyperplasia personal data history for predicting the risk of breast cancer, however this approach has been shown to be ineffective in several populations. Another demonstration by Hart et al. [7] was examined using machine learning algorithm which is capable of predicting the risk of breast cancer and achieved increased prediction accuracy.

Belsare et al. [8] describes a computer-aided concept system that uses a space-color partition of graphical illustrations, tissue property extraction using tools like the gray-level co-occurrence matrix (GLCM), grassland-based ruminant livestock models (GRLM), and Euler methods, and classification using linear discriminative analysis. With 100% accuracy, the algorithm classified 70 histopathology and mammography pictures. Vu et al. [9] employed a feature-oriented vocabulary learning system used on a data set of human intraocular lesions and animal diagnostic laboratories to reach an accuracy of 97.75%. It has been demonstrated that identifying histopathological and mammographic data sets from 70 photos using a computer-aided design technique based on the extraction of morphological characteristics is 85.7% accurate [10]. Mouelhi et al. [11] employed enhanced morphology and adaptive local thresholding algorithms to extract and segment mammography and histochemistry pictures and achieved 98% accuracy.

Khalilabad et al. [12] developed an automated system to analyze how to identify breast cancer masses using micro array pictures into three-fold of preprocessing of images, data mining and detection of the disease and achieved accuracy of 95.45%. Kaymak et al. [13] proposed back propagation neural network as a tool for analyzing and diagnosing breast cancer masses and further improved the technique with radial basis neural network and achieved 59% for the former and 70.4% for the latter. An evolutionary state of the art decision-making approach based on regression and evolutionary approaches has been utilized by Wang et al. [14] to identify breast cancer from mammograms. Mohebian et al. [15] presents a computer-aided diagnostic method for the prediction of breast cancer recurrence that uses optimal ensemble learning or a hybrid approach.

However, researchers highly respect deep learning-based feature extraction techniques for categorizing mammography and histopathology pictures. Also, it is said to outperformed older feature-engineered histopathology analysis techniques in spotting breast cancer metastases in lymph nodes, displaying extremely skilled performance [16]. Wang et al. [17] looked at the usage of an extreme learning machine using deep convolution features for breast cancer diagnosis and classification and achieved remarkable performance of 98.18% classification accuracy. Kumar et al. [18] introduced canine mammary tumors dataset and employed a framework based on VGGNet with different classifiers and achieved mean accuracy of 93%. A support vector machine approach and a deep neural network were combined by Kaur et al. [19] with the goal of categorizing mammographic pictures to find cancerous tumors. This method’s accuracy ranged from 94% to 98%, depending on the different data sets.

Ting et al. [20] proposed a convolutional neural network, CNN-based algorithm for the improvement of breast cancer classification and achieved sensitivity, accuracy and specificity of 89.47%, 90.50% and 90.71% respectively. Li et al. [21] employed an effective and accurate classification of benign and malignant mammography images using an improved DenseNet II model achieving average accuracy of 94.55%. Shen et al. [22] developed a deep neural network algorithm to accurately detect breast cancer on screening mammograms achieving sensitivity and specificity of 86.7% and 96.1% respectively. Saha et al. [23] employed semantic segmentation and classification of breast cancer masses. In this technique, cell membranes and nuclei in the breast region are segmented and categorized using deep learning algorithms such as the human epidermal growth factor receptor-2 deep neural network (Her2Net) and trapezoidal long short-term memory (TLSTM) and achieved accuracy rate of 98.3%.

Rustam et al. [24] used different evaluation metrics like accuracy, sensitivity, specificity, and F1-score to compare linear discriminant analysis with support vector machine (SVM). The outcome demonstrates that the DVM outperforms the linear discriminant analysis in terms of overall performance with 98.8% accuracy. Khan et al. [25] utilized different pre-trained CNNs for the low level features which are fed into a fully connected layer using average pooling for the recognition of malignant and benign cells. Although several deep learning models have been proposed for breast cancer classification and identification, the computational cost and model complexity that may affect the efficacy of breast cancer classification have not been explored thoroughly. Spanhol et al. [26] analyzed six different feature descriptors with several classifiers and achieved 80% to 85% within the magnification factors. In similarity to Spanhol et al. [26], Bayramoglu et al. [27] used an independent magnification factors for the classification of breast cancer and achieved good result. Spanhol et al. [28] presented random-patches of the images for training and testing and achieved an increment of 84% to 91%. Han et al. [29] explored different deep learning models and achieved an average accuracy of 93.2% for patient-level BC classification. Alom et al. [30] proposed an adaptive sparse support vector machine with L1 weighted norm achieving accuracy of 94.97% for 40x magnification factor. A lightweight convolution network is suggested for the identification of breast cancer in line with this perspective.

This paper focuses on the downside of model complexity and computational cost. The novelty of the proposed model is the parallel stacking of separable convolution layers with multiple filters of different sizes with bottleneck convolution layers to shrink feature maps as a technique to reduce dimensionality. The public dataset belonging to Kaggle is used to evaluate the performance of this research.

## 3. Materials and Methods

Data acquisition, data pre-processing, and network training and testing are the phases of the proposed technique. Each step of the proposed approach is detailed in the subsequent headings.

### 3.1. Datasets

**Figure 1:**
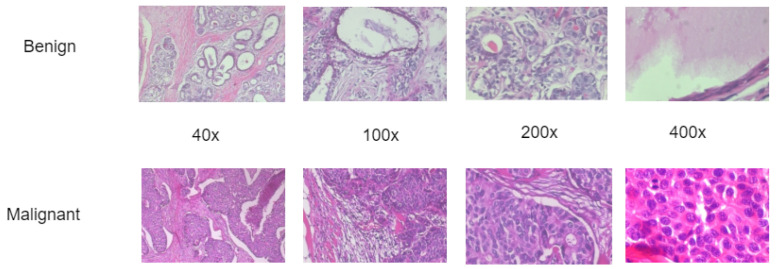
Illustration of the histopathological images of breast cancer from the BreaKHis database.

BreaKHis dataset is an open-source dataset obtained from Kaggle repository to trained the proposed lightweight convolution network. Figure 1 shows both the benign and malignant BreakHis images with different magnifying factors. The BreakHis dataset consists of microscopic histopathological images of breast cancer composed of over 2400 benign and 5400 malignant samples obtained from over 80 patients utilizing different magnifying factors (40×, 100×, 200×, and 400×). The image is 3-channel RGB of 8-bit depth with the resolution of 700×460 pixels in PNG format [26]. Table 1 shows the dataset distribution based on the binary class and magnifying factors while Table 2 shows the multi-class description of the BreakHis dataset.

### 3.2. Data Pre-Processing

**Figure 2:**
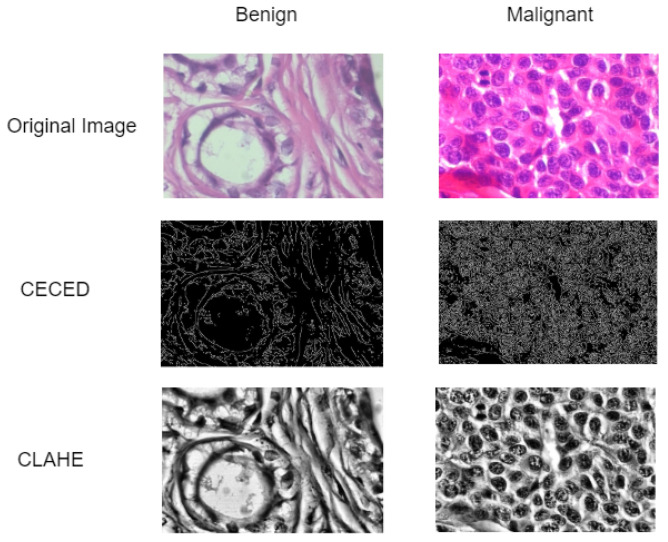
Histopathological breast cancer images. The first row represents the original images. The second row depicts the edge detected images. The third row presents the contrast-enhanced images.

Data pre-processing is a technique often used by deep learning practitioners to enhance the visual characteristics of images. This article adopts contrast enhancement and edge detection techniques in order to enhance the visual trainable features of the histopathological breast cancer images as presented in Figure 2.

#### Contrast Enhancement (CE) and Edge Detection (ED) Images

This paper employed both contrast enhancement [31] and edge detection [32] pre-processing techniques for a better enhancement of the images. First, the application of contrast enhancement makes it more realistic in appearance amongst its histogram equalisation-based member as displayed in Figure 2. In contrast limited histogram equalization, the histogram is cut at some threshold and then equalization is applied. The contrast of an image is enhanced by applying contrast algorithm on small regions called tiles rather than the entire image. The resulting neighboring tiles are then stitched back seamlessly using bilateral interpolation. The contrast in the homogeneous region can be limited so that noise amplification can be avoided. Uniform distribution is used as the basis for creating the contrast transform function. the expression of the modified chrominance channel tile with uniform distribution is given in Equation (1)

(1) $$I_{c\_out}=\left[I_{c\_max}-I_{c\_min}\right]*K_{j}\left(I_{c\_in}\right)+I_{c\_min}$$

where $I_{c\_min}$ and $I_{c\_max}$ are the minimum and maximum permissible intensity levels respectively. $K_{j}\left(I_{c\_in}\right)$ is the cumulative distribution function for input contextual tile $I_{c\_in}$.

The edge detection technique applies Gaussian smoothing for noise reduction while enhancing the morphological image contrast and from the determination of the intensity of the image, hysteresis thresholding is apply for the detection of the edges from the image as depicted in Figure 2. Additionally, less important regions are discarded so the visual characteristics can be detected. The image *f* is first smoothed with a Gaussian filter to reduce noise. This is done by using a convolution with a Gaussian kernel *g* to obtain an image z=f×g. The gradient of the image is calculated in terms of amplitude and angle as seen in Equations (2) and (3). Non-maxima are removed from the amplitude. This means that excessively large outlines are replaced by thinner outlines.

(2) $$M=\sqrt{{\left(h_{x}*z\right)}^{2}+{\left(h_{y}*z\right)}^{2}}$$

(3) $$A=(\frac{h_{y}*z}{h_{x}*z})$$

where $h_{x}$ and $h_{y}$ represents the pixels in *M* which is the gradient of the image.

### 3.3. Lightweight Separable Convolution

**Figure 3:**
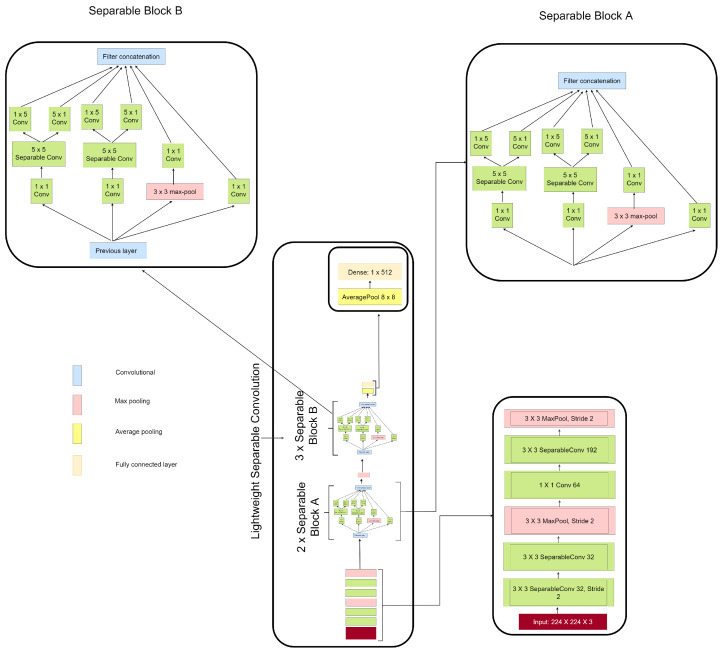
Structure of the lightweight separable convolution.

The novelty behind the proposed method in this paper is in two phases. The first phase involves the implementation of separable convolution layers stacked in parallel with multiple filters of different sizes in order to obtain wider receptive fields as well as achieving wider rather than deeper network. The second phase involves the factorization of convolution layers and the utilization of bottleneck convolution layers to reduce model dimensionality. These methods sufficiently decrease the amount of trainable parameters as well as the computational cost, allowing the network to be developed much deeper and with greater non-linear expressive capacity than plain convolution networks. The proposed architecture consists of three parts, the ‘input head’, ‘separable block layer’, and ‘classification head’. The input head consists of two separable convolution layers followed by a max pool layer before a bottleneck convolution layer and then a separable convolution layer followed by a max pool layer. The separable block layer is divided into ‘Block A’ and ‘Block B’ stacked on top of one another. On the one hand, ‘Block A’ consists of separable convolution layers of 3×3 with bottleneck layers and factorization layers of 1×3 and 3×1 kernel sizes. On the other hand, ’Block B’ consists of separable convolution layers of 5×5 with bottleneck layers and factorization layers of 1×5 and 5×1 kernel sizes as presented in Figure 3. Each separable block has 10 convolution layers and 1 max pooling layer. The proposed method reduced computational cost and achieved reduction in feature dimensionality during the low-level feature extraction and overall network depth. In the classification head, the regular fully connected layer is replaced with average pooling of 8×8 to flatten the feature vector and finally, one dense layer having its dimension set as 1×512 is adopted as seen in Figure 3. To overcome over-fitting, dropout layers were added.

## 4. Experimental Results

This section presents the evaluation results of the proposed architecture both on binary and multi-class categories. The effect of data pre-processing on the performance of the proposed model is also detailed in this section. The standard metrics utilized to examine the diagnostic performance of the proposed LWSC are specificity (SPE), accuracy (ACC), precision (PRE), and sensitivity (SEN). The numerical expression for each metric is presented in Equations (4)–(8) [13,14].

(4) $$Accuracy=\frac{TP+TN}{TP+TN+FP+FN}$$

(5) $$Sensitivity=\frac{TP}{TP+FN}$$

(6) $$Specificity=\frac{TN}{TN+FP}$$

(7) $$Precision=\frac{TP}{TP+FP}$$

(8) $$F1-score=2*\frac{Precision*Recall}{Precision+Recall}$$

*TN* denotes true negative, *TP* stands for true positive, *FP* depicts false positive and *FN* denotes false negative.

### Evaluation of the Lightweight Separable Convolution

**Figure 4:**
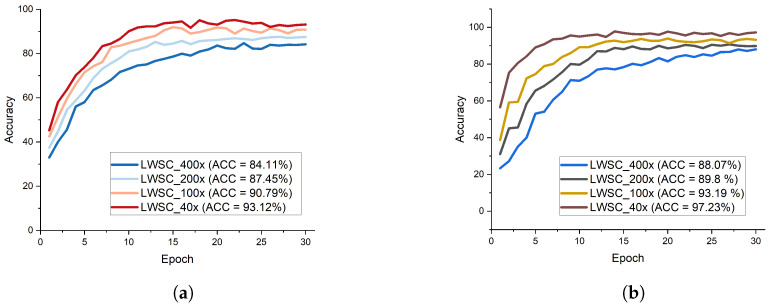
Accuracy performance report of the BC classification for both two classes and eight classes. (**a**) Test accuracy curve for different magnification factors for two classes of BC classification using the proposed model with contrast-enhanced images, (**b**) Test accuracy curve for different magnification factors for eight classes of BC classification using the proposed model with contrast-enhanced images.

Extensive study was conducted to evaluate the influence of the proposed lightweight separable convolution network to the identification performance in terms of accuracy on histopathological breast cancer benchmark dataset known as BreakHis. The first study considered the original BreakHis images for the identification of histopathological breast cancer. The second study considered the edge enhanced BreakHis images for the identification of histopathological breast cancerand finally, the third study considered the contrast enhanced BreakHis images for the identification of histopathological breast cancer. The proposed LWSC model presented in Figure 3 clearly revealed that the multiple receptive fields is capable of handling low quality images in histopathological breast cancer identification obtaining better recognition performance on both binary and multi-class categories. Figure 4a shows the test accuracy curves for the different magnifying factors on the binary category indicating that the model obtains high accuracy of 93.12% on the 40× magnifying factor of the contrast enhanced image while Figure 4b represents the test accuracy curves for the different magnifying factors on the multi-class category indicating that the model obtains high accuracy of 97.23% on the 40× magnifying factor of the contrast enhanced image.

Table 3 illustrates the recognition accuracy for the original raw image and the pre-processed images for both contrast and edge enhancements. The LWSC + CE-based images denotes the contrast enhanced image with LWSC, the LWSC + ED-based images denotes the edge enhanced image with LWSC, and the LWSC + Original images denotes the raw original image with LWSC for the identification of histopathological breast cancer. From all indications, the proposed lightweight separable convolution model with contrast enhanced image outweighs both the original raw and the edge enhanced images achieving 93.12% accuracy on the binary class category of magnifying factor of 40× and 97.23% accuracy on the eight class category of magnifying factor of 40×. The proposed LWSC with CE outperforms the other combinations on all evaluation metrics.

The classification performance of LWSC with contrast enhanced image is higher than that of edge enhanced image which suggest that the contribution of contrast enhanced histopathological images in breast cancer identification is greater than that of edge enhanced histopathological images. The proposed LWSC is further evaluated in terms of SPE, SEN, PRE, and AUC on both categories of class labels with the magnifying factor of 40× as depicted in Table 3. It is observed that the proposed LWSC performs better on multi-class category with the magnifying factor of 40× achieving 97.23% accuracy, 97.71% sensitivity, 97.93% specificity, and 98.11% precision.

## 5. Discussion

The efficacy of the proposed method in identifying breast cancer DR in histopathological images with different magnifying factors on both binary and multi-class categories has been presented and the classification result is presented in Table 3. As denoted by the above mentioned results, the proposed LWSC can efficiently classify the different breast cancer types for the multi-class category. It is important to note that the proposed LWSC indicates better generalization ability with the contrast enhanced histopathological images with a commendable computational efficiency of 5.9 min training time. The proposed method is further compared with some up-to-date methods using BreakHis dataset and other benchmark dataset. Table 4 indicates that the proposed LWSC obtains satisfactory performance in sensitivity, specificity, and F1 score of 97.71%, 97.93%, and 97.98% respectively. Rustam et al. [24] achieved the highest accuracy value of 98.77%. Kaur et al. [19] obtained the highest AUC value of 99.0%.

The proposed LWSC obtains the highest accuracy score of 97.23% indicating the superiority of the proposed method for histopathological breast cancer identification. The competitive merit of the proposed model is attributed to the wider receptive fields from the different filter sizes. It is well known that different deep learning architecture will show different behaviours for different conditions. In order to select what number of separable blocks and its combination will produce the best result for the proposed lightweight separable convolution, ablation study is conducted.

Table 5 shows the experimental results obtained by the proposed architecture in comparison with various pre-trained networks on the BreakHis dataset using the same computing device. From the result, VGG-19 model shows better performance, achieving 96.4% accuracy, 97.1% sensitivity, and 96.0% specificity. Inception V3 model indicates significant improvement in performance, achieving 95.8% accuracy, 96.0% sensitivity, and 95.3% specificity.

**Figure 5:**
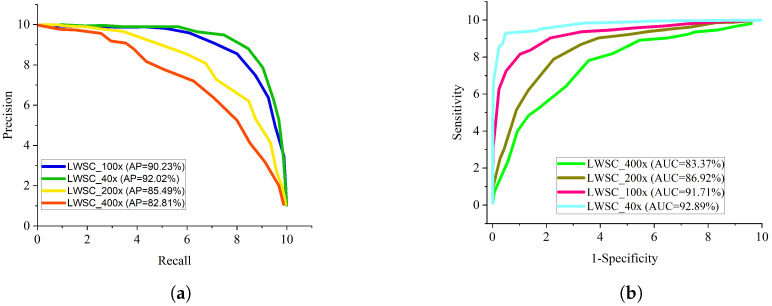
Comparison report of the BC classification for two classes. (**a**) Precision–recall curve for different magnification factors for two classes of BC classification; (**b**) ROC curve for different magnification factors for two classes BC classification.

**Figure 6:**
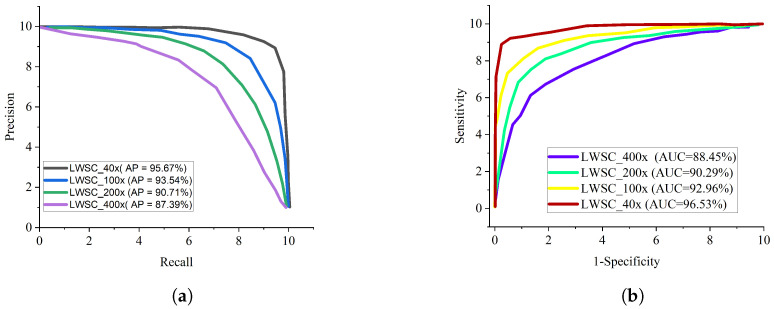
Comparison report of the BC classification for eight classes. (**a**) Precision–recall curve for different magnification factors for eight classes BC classification; (**b**) ROC curve for different magnification factors for eight classes BC classification.

In general, AlexNet gave the least performance across all evaluation metrics followed by Xception model. Considering a sensitive condition like histopathological breast cancer, it is imperative to adopt the ROC curve as a method to examine the total accuracy and the precision-recall curve to examine the average precision of the proposed lightweight separable convolution. The precision-recall curve is presented in Figure 5a while the ROC curve for the proposed LWSC model on binary category is presented in Figure 5b. Similarly, Figure 6a,b present the precision-recall curve and the ROC curve for the proposed LWSC model on multi-class category respectively.It is worthy to mention that the pre-trained models and the proposed LWSC model are trained using the same computing resource and dataset for fair comparison.

Additionally, some of the histopathological images were blurred which could have prevented the proposed LWSC model from training useful features. The advantage of improving the visual trainable features of the histopathological images using contrast enhancement and edge detection pre-processing techniques is to characterize distinctive representation features of the histopathological images with viable trainable details. The proposed LWSC obtained a significant performance in classifying histopathological breast cancer.

From all indications, the proposed LWSC outperforms the other networks in the perspective of precision-recall and ROC especially in handling low quality histopathological images. The precision-recall graphs shows that the curve of the proposed LWSC model is nearest to the upper right corner of the graph which implies that the proposed LWSC model has high precision associated with high sensitivity. Similarly, the ROC graphs depicts that the curve of the proposed model is nearest to the upper left corner of the graph which implies that it has high sensitivity associated with high specificity. Importantly, the obtained result presented by ROC and precision-recall curves can help expert histopathologist in maintaining a balance between accuracy and precision.

This study has achieved a significant degree of accuracy in classifying histopathological breast cancer however, there are certain setbacks. The level of accuracy obtained on histopathological breast cancer dataset might not be the same for another medical dataset. The singular reason is due to the fact that images of different dataset differ owing to different factors such as labeling, noise, image collection method, and location. Aside the non-uniformity of data, the partitioning of the data category is also paramount. The differences in class weight has a negative effect on training. The classification accuracy is also affected by the various data augmentation techniques adopted to correct class weight imbalance. In light of these constraints, studies will be carried out in the future to accommodate a wider range of dataset and possibly utilize different hyper-parameter tuning techniques.

## 6. Conclusions

This manuscript proposed a technique of identifying histopathological breast cancer using lightweight separable convolution neural network trained on BreakHis dataset. Image contrast enhancement and edge detection were implemented as pre-processing steps to extract visual trainable characteristics in order to achieve high classification accuracy. The proposed LWSC model implements a separable convolution layers stacked in parallel with multiple filters of different sizes in order to obtain wider receptive fields as well as achieving wider rather than deeper network. Factorization of convolution layers and the utilization of bottleneck convolution layers to reduce model dimensionality were introduced in the proposed LWSC model. The proposed work sufficiently decrease the amount of trainable parameters as well as the computational cost, allowing the network to be developed much deeper and with greater non-linear expressive capacity than plain convolution networks. The proposed LWSC model outperforms several state-of-the-art models. The evaluation results depict that the proposed LWSC model performs optimally obtaining 93.12% accuracy, 93.61% sensitivity and 94.07% specificity on binary category while on multi-class category, the proposed LWSC obtained 97.23% accuracy, 97.71% sensitivity, and 97.93% specificity. From the comparative results of the other established techniques, it is confirmed that the proposed LWSC model obtained state-of-the-art classification accuracy which makes it an efficient solution for breast cancer diagnosis. These findings could efficiently help expert histopathologist in maintaining a balance between accuracy and precision while saving time.

## Figures and Tables

**Table 1:** Description of the BreakHis dataset for Binary class.

Magnifying Factor	Benign	Malignant	Total
40×	625	1370	1995
100×	644	1437	2081
200×	623	1390	2013
400×	588	1232	1820
Sum Total	2480	5429	7909
Number of Patients	24	58	82

**Table 2:** Description of the BreakHis dataset for Multi-class.

Classes	Sub-Classes	Patients Count	40×	100×	200×	400×	Total
	Adenosis (A)	4	114	113	111	106	444
	Fibroadenoma (F)	10	253	260	264	237	1014
Benign	Tubular Adenoma (TA)	3	109	121	108	115	453
	Phyllodes Tumor (PT)	7	149	150	140	130	569
	Ductal Carcinoma (DC)	38	864	903	896	788	3451
Malignant	Lobular Carcinoma (LC)	5	156	170	163	137	626
	Mucinous Carcinoma (MC)	9	205	222	196	169	792
	Papillary Carcinoma (PC)	6	145	142	135	138	560
Total		82	1995	2081	2013	1820	7909

**Table 3:** Comparison of the proposed model with different pre-processing images.

Model	Binary Class						Multi-Class					
	ACC (%)	SEN (%)	SPE (%)	PRE (%)	AUC (%)	Time (min)	ACC (%)	SEN (%)	SPE (%)	PRE (%)	AUC (%)	Time (min)
LWSC + Original images	90.11	90.07	91.76	91.78	92.45	7.3	92.13	91.78	90.66	91.92	92.41	7.5
LWSC + ED-based images	91.76	92.18	90.82	91.65	92.55	5.8	93.71	92.62	91.09	92.85	93.16	5.9
LWSC + CE-based images	93.12	93.61	94.07	93.34	94.21	5.8	97.23	97.71	97.93	98.11	98.02	5.9

**Table 4:** A comparison analysis of the proposed model with state-of-the-art models on BreakHis dataset.

State-of-the-Art Model	ACC (%)	AUC (%)	SPE (%)	SEN (%)	F1-Score (%)
Kumar et al. [18]	93.0	95.0	NR	NR	94
Kaur et al. [19]	92.0	99.0	90.0	93.0	96.0
Ting et al. [20]	90.50	90.10	90.71	89.4	NR
Rustam et al. [24]	98.77	NR	97.64	96.44	99.0
Spanhol et al. [26]	85.0	86.1	NR	NR	NR
Bayramoglu et al. [27]	83.25	NR	NR	NR	NR
Alom et al. [30]	97.65	98.91	97.52	92.9	NR
Hao et al. [33]	96.75	NR	96.9	97.18	NR
LWSC + CE-based images	97.23	96.53	97.93	97.71	97.98

**Table 5:** Fair comparison of the proposed model on BreakHis dataset using different pre-trained models on the same computing device.

Model	Binary Category			Multi-Class Category		
	ACC	SEN	SPE	ACC	SEN	SPE
AlexNet	89.5	91.6	88.0	88.9	88.0	87.2
VGG-16	95.2	96.3	94.9	96.4	97.1	96.0
ResNet-101	93.9	94.5	94.1	94.5	95.0	93.2
DenseNet-121	94.5	93.7	92.2	94.2	94.6	94.9
Inception V3	97.3	97.8	97.0	95.8	96.0	95.3
Xception	91.9	92.6	89.6	91.3	92.8	90.7
LWSC + CE-based images	93.12	93.61	94.07	97.23	97.71	97.93

## Data Availability

The datasets as cited within the text as reference [26] used in this study are available at: https://www.kaggle.com/datasets/ambarish/breakhis (accessed on: 23 June 2022).
